# The Spanish transplantation model as a benchmark: clinical, societal, and economic impact of liver transplants in Spain (1984–2024)

**DOI:** 10.1186/s13561-026-00801-4

**Published:** 2026-06-02

**Authors:** Edson Plasencia Sánchez, Concepción Gómez Gavara, Glòria Merino Pinto, Roger Sabater Mezquita, Itxarone Bilbao, Gerardo Blanco-Fernández, Gloria de la Rosa, Gemma Piella

**Affiliations:** 1 SIMBIOsys Group, Department of Engineering, Universitat Pompeu Fabra, Carrer de Tanger 122, Barcelona, 08018 Spain; 2 Department of Hepatic and Biliary Surgery and Transplants, Hospital Universitari Vall d’Hebron, Pg. Vall d’Hebron 119, Barcelona, 08035 Spain; 3 Centre de Recerca en Economia i Salut, Departament d’Economia i Empresa, Universitat Pompeu Fabra, Barcelona, 08005 Spain; 4 Department of Econometrics, Statistics and Applied Economics, Universitat de Barcelona, Barcelona, 08007 Spain; 5 Department of Surgery, School of Medicine, Universitat Autònoma de Barcelona, Bellaterra, 08193 Spain; 6 Sociedad Española de Trasplante Hepático (SETH), Madrid, Spain; 7 Servicio de Cirugía Hepatobiliopancreática y Trasplante Hepático, Hospital Universitario de Badajoz, Badajoz, Spain; 8 Organización Nacional de Trasplantes (ONT), Ministerio de Sanidad, Madrid, Spain

**Keywords:** Liver transplantation, Societal value, Economic impact, Health Economics, Spain

## Abstract

Spain leads the world in organ donation, and the 40th anniversary of its first liver transplant was recently commemorated. Our aim was to evaluate the evolution of the Liver Transplantation (LT) program in Spain over its four decades (1984–2024), examining clinical outcomes and societal economic benefits within a mature transplantation framework. Standardized national data from the *Organización Nacional de Trasplantes* and the *Sociedad Española de Trasplante Hepático* were used to reconstruct recipient cohorts by age group. Patient survival was estimated using Kaplan–Meier analysis. An economic evaluation from a societal perspective integrated surgical costs, long-term immunosuppressive therapy, and productivity gains associated with recipients’ participation in the labour market. LT activity increased steadily during the initial expansion of the program and has stabilised at approximately 1,200 procedures per year over the past two decades. Both donor and recipient populations have aged; there are currently more patients over 60 years of age than two decades ago. Survival improved throughout the study period, extending life expectancy despite variations related to underlying disease. From an economic perspective, annual surgical costs remained below €60 million, increasing to €70–90 million when long-term immunosuppressive therapy was included. The economic contribution of surviving recipients to the labour market increased steadily, exceeding €75 million in 2024 and resulting in a positive balance since the early 2010s. Adult recipients generated net economic benefits, whereas paediatric recipients showed delayed but progressively increasing contributions as they entered the workforce. LT in Spain has produced sustained improvements in expected survival while generating substantial societal value, demonstrating that LT is both an effective clinical intervention and a valuable public health investment. The Spanish liver transplantation system may serve as a benchmark for evaluating the sustainability and societal impact of liver transplant programs in other healthcare systems.

**Supplementary Information:**

The online version contains supplementary material available at 10.1186/s13561-026-00801-4.

## Introduction

Liver transplantation (LT) has evolved into a life-saving therapy for patients with end-stage liver disease, acute liver failure, and certain hepatic malignancies [1]. Since the first successful LT in the 1960 s, advances in surgical techniques, immunosuppressive therapies, and perioperative care have dramatically improved patient survival and graft longevity [2, 3].

Beyond its clinical life-saving impact, LT carries substantial economic value. By preventing long-term complications, reducing hospitalizations, and enabling patients to return to work and/or resume family caregiving roles, transplantation represents a valuable investment in public health. Quantifying the economic impact of LT is therefore essential to inform policy decisions, optimize resource allocation, and ensure the sustainability of transplant systems. Comprehensive economic assessments should consider the full continuum of care, including pretransplant evaluation, the transplantation procedure, and post-transplant follow-up. Early studies in the 1990 s focused primarily on the direct costs of LT [4–6], while subsequent analyses examined the economic burden of liver diseases or evaluated the cost-effectiveness of treatments and screening strategies [7–11]. Nonetheless, data on the costs associated with patients on transplant waiting lists, complications of chronic liver disease, and long-term post-transplant management remain limited [12–15]. Addressing these gaps is crucial for guiding policy, optimizing resource allocation, and maximizing both clinical and economic benefits.

In this global context, Spain stands out as a leading example in organ donation and transplantation. In 2024, Spain reported 52.6 donors per million population, ranking among the highest in Europe and consolidating its world leadership in organ donation [16, 17]. Over the past four decades, most LT procedures have been performed with deceased donors, and although some have involved living donors, especially for younger recipients, these are less common. This success reflects a highly organized transplantation system, supported by a national coordination network, robust organ donation programs, and comprehensive follow-up protocols. These efforts have not only increased access to LT but also enhanced equity, efficiency, and patient outcomes, establishing Spain as a global benchmark in donation rates and transplant success.

Despite these achievements, LT continues to face challenges, including organ scarcity, increasingly complex recipients, and the need to optimize long-term graft and patient survival [1, 18]. Evolving indications, technological innovations, and public health dynamics further underscore the necessity of periodically evaluating past experiences to guide future strategies and economic planning.

This paper provides a comprehensive overview of 40 years of LT in Spain, highlighting historical milestones, clinical and organizational advancements, and current outcomes. By synthesizing clinical, economic, and societal data, our framework reveals a macro-level impact that remains obscured when these components are evaluated as isolated metrics. To our knowledge, this represents the first national-level synthesis to offer a longitudinal perspective on how transplant activity translates into sustained societal value, providing insights to support future policy and clinical practice.

## Methodology

The analysis is based on public available data extracted from the annual reports of the Spanish Liver Transplant Registry (*Registro Español de Trasplante Hepático*, RETH), compiled jointly by the Spanish National Transplant Organization (*Organización Nacional de Trasplantes*, ONT) and the Spanish Liver Transplant Society (*Sociedad Española de Trasplante Hepático*, SETH) [16]. All data processing, analyses, and interpretations were conducted independently by the authors.

### Recipients series

We consolidated and standardized records from RETH annual summaries [16] by converting multi-year aggregate data into counts to construct a continuous annual time series of the number of LT recipients spanning the entire 40-year period from 1984 to 2024. Following the most common criteria used in RETH annual summaries, recipients were categorized into four age groups: Group 1 (0–15 years), Group 2 (16–39 years), Group 3 (40–59 years), and Group 4 (≥ 60 years).

Group 1 corresponds to paediatric recipients, treated in specialised transplant centres. The number of recipients for this group was available in RETH annual summaries for all years except 1992–1993, 2007–2012 and 2017–2018. For these years, estimates were derived from their percentage reported in a multi-annual basis in the RETH annual summaries. For Groups 2–4, annual counts were estimated by multiplying their corresponding percentages by the difference between the total number of recipients and those previously obtained for Group 1. To avoid improbable fluctuations in recipient numbers while preserving the total annual count and the multi-annual proportion, imputations were applied to Group 2 and Group 3 in 1986 and 1989, and to Group 4 in 1996 and 1997. Finally, minor adjustments (± 1 recipient) were made to Group 4 counts when necessary to correct for rounding effects introduced by converting multi-annual percentage distributions into number of recipients.

### Time series of surviving recipients

Kaplan–Meier charts in the RETH annual summaries [16] provide the percentage of recipients that survive over time. According to the time evolution of surviving recipients by each diagnosed pathology (see details in Figure A1), both paediatric and adult recipients were grouped in two categories: those with higher life expectancy (life expectancy 1, LE1) and those with lower life expectancy (life expectancy 2, LE2). Paediatric recipients with LE1 included those diagnosed with cholestasis or metabolic disease (excluding hepatocellular carcinoma), while paediatric recipients with LE2 comprise all those diagnosed with hepatocellular carcinoma, cirrhosis without hepatocellular carcinoma or acute liver failure. In contrast, adults with LE1 are those diagnosed with cholestasis, metabolic disease (excluding hepatocellular carcinoma) or acute liver failure, while adults with LE2 included those diagnosed with hepatocellular carcinoma or cirrhosis without hepatocellular carcinoma.

Table 1 summarises the survival patterns for LE1 and LE2 recipients between 1991 and 2023, while detailed information is provided in Supplementary Information (Tables A1 and A2). Differences in long-term survival between the two groups are more pronounced among paediatric recipients, and overall survival increases with the year of LT (see Section III-4 for more details); in the LE1 group, survival declines from 88% at 1 year to 73% at 32 years post-transplant (covering the full follow-up period from 1991 to 2023), whereas in the LE2 group, survival decreases from 75 to 45% over the same period. The time series of surviving recipients were constructed by multiplying the corresponding survival probability (of either LE1 or LE2) by the number of transplants performed in each preceding year over the period 1991–2022 (corresponding to lags of up to 32 years) for both paediatric and adult recipients.

**Table 1:** Recipient life expectancy for transplants performed between 1991–2023, expressed as the percentage of surviving recipients at selected time horizons following liver transplantation, stratified by life expectancy group (LE1 and LE2) and age category (paediatric: ≤ 16 years; adult: > 16 years)

Life expectancy		1y	3y	6y	9y	12y	15y	20y
LE1	Paediatric	87.8%	85.7%	82.5%	80.0%	80.0%	77.6%	72.5%
	Adult	93.0%	86.7%	82.6%	77.7%	72.8%	66.9%	58.0%
LE2	Paediatric	74.7%	60.6%	60.0%	55.0%	45.0%	45.9%	45.0%
	Adult	87.4%	81.2%	74.1%	66.5%	59.0%	51.7%	40.0%

### Costs associated with recipients

The economic valuation of LT was estimated by considering both the costs associated with the number of LT performed in a given year and those associated with the number of surviving recipients in that year. The first component corresponds to the surgical procedure, while the second component reflects the annual expenses related to post-transplant clinical follow-up and immunosuppressive therapy for surviving recipients. All monetary values were standardised to 2023 prices using the inflation rates stated by the National Statistics Institute [19].

The cost of a LT in Spain was obtained from estimates provided by the Spanish Ministry of Health, which reported a mean cost of €53 thousand (standard deviation 39 thousand, 95% confidence interval: €51–56 thousand) per surgical procedure in 2019 [20], corresponding to €55 thousand in 2023 after adjusting for inflation. The immunosuppressive therapy cost per surviving recipient was estimated at €2.2 thousand per year in 2023 (€2 thousand on average during the last 20 years) according to official reference prices for medicines [21]. In this study, certain cost components (e.g., hospital readmissions, lifelong follow-up by hospital specialists and primary care providers) were not included due to their lower frequency and limited contribution to the overall costs. For instance, the readmission rate for diseases and disorders of the liver, biliary system, and pancreas changed only slightly from 9.5% to 10.5%, between 2009 and 2021, while the average length of stay reduced from 8 to 6 days [22], and the number of retransplants has remained below 10% for the last 25 years (see Figure A2). Therefore, the annual total cost of LT in Spain was calculated by summing the surgical procedure costs for all LTs performed and the immunosuppressive treatment costs for surviving recipients. Mathematically, this is expressed as:

1 $$AnnualLTCost\left(t\right)=\sum\limits_{i=1}^{4}{N}_{i}\left(t\right)\bullet {Cost}_{Tx}+\sum\limits_{i=1}^{4}\sum\limits_{k=1984}^{t-1}{SR}_{i}(k,t) \bullet {Cost}_{Immuno}$$

where, for year t = 1984,…, 2024, ${N}_{i}\left(t\right)$ represents the number of LTs performed in i-*th* age group, ${Cost}_{Tx}$ is the cost of the surgical procedure in 2023, ${Cost}_{Immuno}$ is the cost of immunosuppressive therapy per surviving recipient in 2023, and ${SR}_{i}(k, t)$ represents the number of surviving recipients transplanted in year k who are alive in year t in the i-*th* age group. For *t=1984*, the second term is zero because the summation over *k* is empty, and survival rates are calculated on an annual basis as mentioned in section II.2.

### Economic incomes associated with recipients

Each adult recipient who returns to their productive activity after LT contributes indirectly to amortize their own treatment costs, and over time the productivity of all recipients returning to activity could make the LT activity self-sustainable in these terms. In addition, paediatric recipients who reach an age sufficient to enter the labour market will produce income that would not otherwise exist. Studies in the United States (US) and Italy have found that between 40–50% of adult LT recipients were retired or did not return to work [23, 24], while at least 30% of paediatric LT recipients obtained employment and remained active 20 years after LT. Between 20 and 50% of both paediatric and adult surviving recipients achieve higher educational levels, are employed, and are married [24, 25]; therefore, it is reasonable to infer that a similar proportion of surviving LT recipients in Spain participate in the labour market. This assumption is supported by comparable findings from single-center studies in Spain [26] and Italy [23], as well as by demographic and clinical similarities in LT programs between the two countries. Based on this evidence, we assume that a similar fraction of LT recipients in Spain is able to return to work. Accordingly, a return-to-work (RW) ratio of 0.5 [23, 24] is assumed for Groups 2, 3 and 4, such that we assume that half of the transplanted individuals in each group will be employed. Recipients in Group 1 are outside the labour market (RW = 0) before entering Group 2. Data from Surveys of the Working Population (*Encuestas a la Población Activa*, EPA) [27] show that only 1% of the population in Group 4 remain generating economic incomes over 70. To account for heterogeneity in labour market participation between groups, an active population (AP) factor (i.e. the ratio of active population to the total population) is considered, according to historical EPA time series. Mean income per age group, derived from EPA time series and adjusted for inflation to 2023, is then used to estimate the annual income associated with LT recipients. Specifically, aggregate annual income is calculated by multiplying the number of LTs by the corresponding RW ratio, the AP factor, and the MI for each age group:

2 $${Annual Income}_{Tx}\left(t\right)=\sum\limits_{i=1}^{4}\left[{N}_{i}\left(t\right)\bullet {AP}_{i}\left(t\right)\bullet {MI}_{i}\left(t\right)+\sum\limits_{k=1984}^{t-1}{SR}_{i}\left(k,t\right)\bullet {AP}_{i}\left(t\right)\bullet {MI}_{i}\left(t\right)\right]\bullet {RW}_{i}$$

where, for year t = 1984, …, 2024, ${N}_{i}\left(t\right)$, ${AP}_{i}\left(t\right),$ and ${MI}_{i}\left(t\right)$ represent, respectively, the number of LT recipients, the active population factor, and the Spanish mean income according to historical EPA time series [27] and standardised to 2023 prices [19] in i*-th* age group (< 16, 16–39, 40–59 and ≥ 60 years). As with the previous equation, ${SR}_{i}(k, t)$ denotes the number of surviving recipients transplanted in year k who are alive in year t. RW_i_ is the specified return-to-work ratio of 0.5.

### Life-years gained

Life-Years Gained (LYG) per recipient is a measure that quantifies the additional number of years of life that a person is expected to live as a result of receiving a health treatment [28]. Mathematically, it is defined as:

$$LYG(k,T)= \int\limits_{k}^{T}\left({S}_{1}(t)-{S}_{0}(t)\right)dt$$

where S_1_(t) and S_0_(t) are the surviving probabilities at year t over a time horizon T-k with health intervention (transplantation, with subscript 1) and without intervention (no transplantation, with subscript 0), respectively.

Because patients in the waiting lists are prioritised according to the Model for End-Stage Liver Disease (MELD), the probability that LT recipients survive more than 3 months without transplantation is below 50%. Consequently, S_0_(t) is assumed to be negligible over the horizon of years considered, so S_0_(t) is set equal to 0. Under this assumption, the LYG per recipient corresponds to the area under the curves in the Kaplan–Meier charts for each group. Since each patient contributes one life-year for each year they survive, the total LYG can be approximated by summing surviving recipients across all years of follow-up:

3 $$Total LYG=\sum\limits_{i=1}^{4}\sum\limits_{t=1984}^{2024}\sum\limits_{k=1984}^{t}S{R}_{i}\left(k,t\right)$$

where, again, $S{R}_{i}(k,t)$ is the number of surviving recipients transplanted in year k who are alive in year t.

## Results

### Evolution of LT activity

**Fig. 1:**
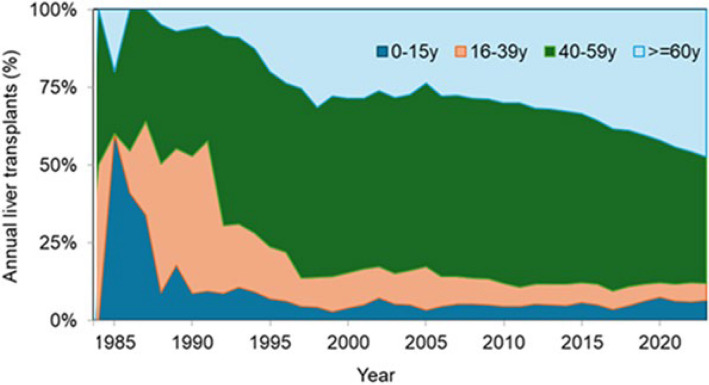
Changes in the liver recipients profile according to age category (in years). Percentages calculated from the annual summaries data of the *Registro Español de Trasplante Hepático* (Spanish Liver Transplant Registry, RETH) [17]

During the 1980 s, only a few dozen LTs were performed annually. Since then, the number of recipients has increased steadily, stabilising over the past two decades at approximately 1,200 per year. The distribution across recipient age groups has changed over time. Group 1 and 2 declined during the first two decades of LT activity but have since stabilised at approximately 5% each (60–80 recipients per year). Group 3 has historically represented the largest proportion of recipients (600–680 recipients per year), but its share has fallen to nearly 50% in recent years (640–470), losing ground to Group 4. Undoubtedly, Group 4 has grown the most (starting with fewer than 300 recipients per year before 2000, it reached 390 in 2015 and more than 500 in recent years), mainly due to the increase in life expectancy among the population and the availability of donors of such group age in Spain. Fig. 1 shows the evolution of recipient group distribution over the entire period of LT activity in Spain.

### Survival and life expectancy of recipients

**Fig. 2:**
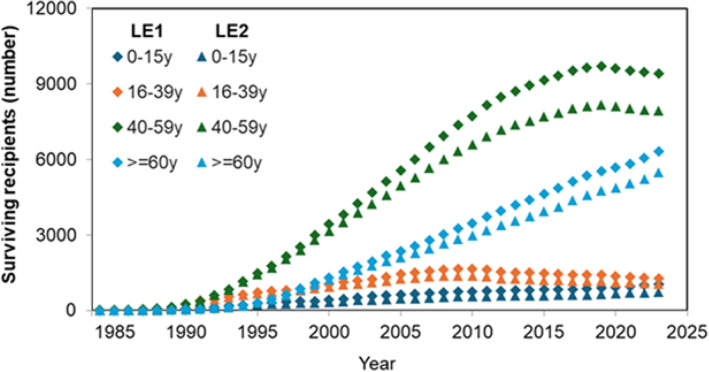
Evolution of the number of surviving recipients according to age category (in years). Estimated from Kaplan–Meier charts and data published in the annual summaries of the *Registro Español de Trasplante Hepático* (Spanish Liver Transplant Registry, RETH) [17]

Figure 2 illustrates the evolution of surviving recipients both under the best-case and the worst-case life expectancy scenario (LE1 and LE2) by age group. In general, the number of surviving recipients exhibits an upward trend, roughly doubling the number of transplants per year. This behaviour reflects the advances in donor and recipient selection, along with improved knowledge of transplantation techniques and clinical case management, which have positively influenced the life expectancy of recipients over time.

As in Spain, improvements in short-term survival among LT recipients have also been observed since the early 1980 s in countries such as the US [29] and Japan [30]. Long-term outcomes, however, show mixed trends: among larger recipient populations in other countries with heterogeneous treatments, no statistically significant changes have been reported [29], whereas smaller recipient populations with similar treatments have experienced improvements over time [30]. Spain appears to follow the latter trend according to RETH annual summaries.

### Economic impact of LT activity

**Fig. 3:**
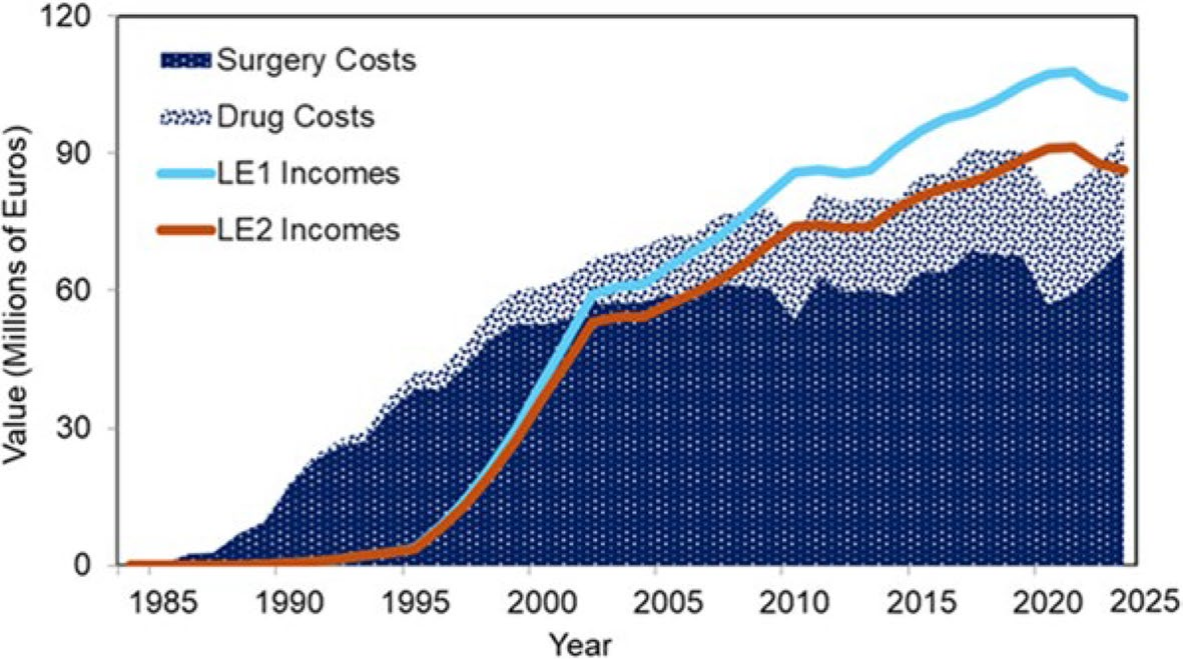
Economic balance of liver transplants since 1984. The dotted area represents total cost, including surgery (darker) and drug treatment expenditures (lighter). The light blue and orange lines represent the estimated contribution to mean income from surviving recipients with LE1 and LE2 respectively, calculated based on historical EPA time series of mean annual incomes for the active population [27] and assuming a 50% of returning work ratio. Costs obtained from *Ministerio de Sanidad* (Spanish Ministry of Health) [20, 21]

The economic impact of LT has evolved considerably over time. Fig. 3 illustrates the economic balance of LT since 1984. Total costs (surgery and treatment expenditures) are shown as dotted blue areas, while the estimated economic contribution of active surviving recipients is represented by coloured lines. This economic contribution was calculated based on the historical time series of annual mean incomes per age group, adjusted for the factor of active population and assuming a 50% RW ratio. The contribution of adult surviving recipients to the active economy, which was negligible before 1990, rose continuously at an accelerating pace until approximately 2002. Thereafter, growth persisted but at a slower rate, reaching approximately €87 million in 2023. On the other hand, costs increased steadily throughout the 1980 s and 1990 s, reaching around €50 million by the early 2000 s, and stabilised between €70–80 million as the annual number of recipients plateaued at about 1,100–1,200 cases from 2010 to 2020. Since then, income has remained very close to total costs, but always above the surgery costs and well differentiated according to life expectancy scenarios (LE1 or LE2).

**Fig. 4:**
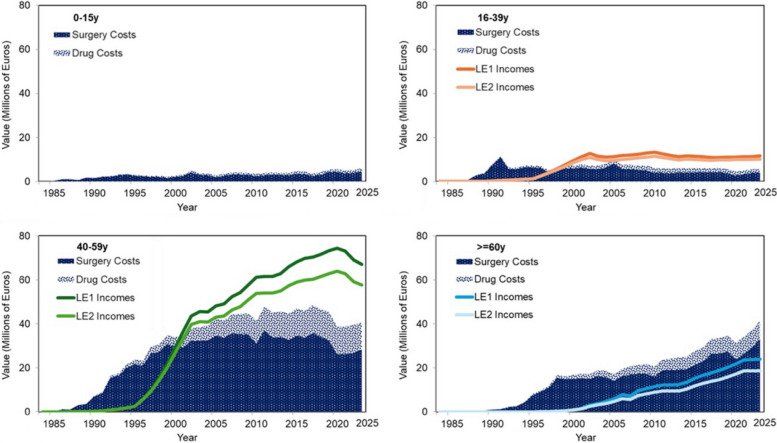
Economic balance by age group. Plots are arranged from left to right and top to bottom: Group 1 (0-15y), Group 2 (16-39y), Group 3 (40-59y), and Group 4 (≥ 60y). Costs obtained from *Ministerio de Sanidad* (Spanish Ministry of Health) [20, 21] and Incomes from EPA historical time series [27]

When stratifying the net economic balance by age group and tracking outcomes over a 20-year follow-up period, Group 1 and Group 4 exhibited a negative balance, with costs exceeding the corresponding mean income for their active population. This finding is explained by both the low economic activity in the older population (Group 4) and the fact that there is no economically active population in Group 1. In contrast, all other groups reached a positive balance, with Group 2 achieving it as early as 1997 and Group 3 by 2003 (Fig. 4).

### Non-economic impact of LT activity

**Fig. 5:**
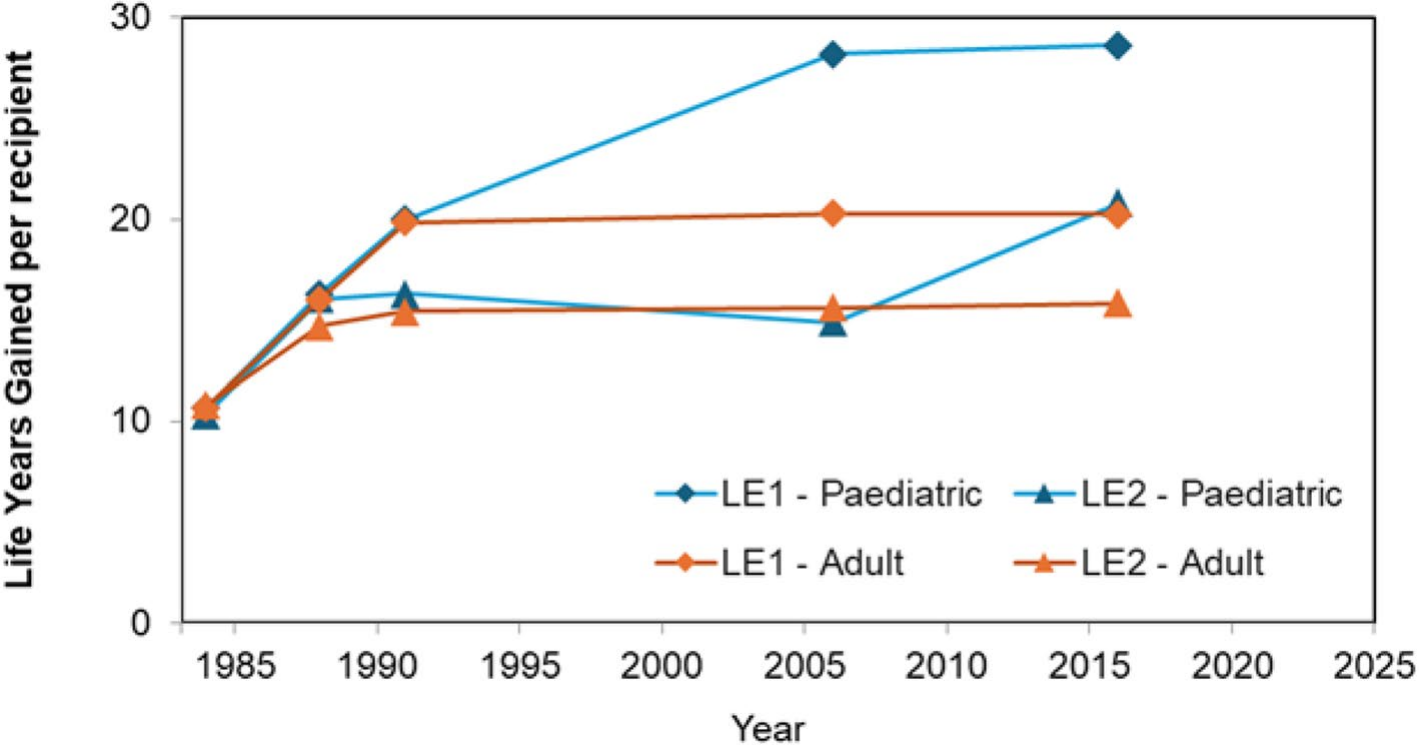
Evolution of Life Years Gained over 1984–2023. Estimated from Kaplan–Meier charts and data published in the annual summaries of the *Registro Español de Trasplante Hepático* (Spanish Liver Transplant Registry, RETH) [17]

In addition to the economic aspect, the number of Life-Years Gained (LYG) due to LT has increased steadily since the early 1980 s, particularly for the paediatric population (Group 1), who nowadays gain between 21 (LE2) and 29 (LE1) years. Adult recipients gain on average between 16 and 20 years (see Fig. 5). Some recipients, however, have lived much longer; for example, a famous Spanish singer remained economically active well into his 80 s [31], as well as international athletes and businessmen into their 60 s and 70 s [32, 33]. These examples illustrate that LT not only extends life but also enables recipients to maintain high levels of functioning, productivity, and social engagement.

Figure 5 also provides an additional socio-economic argument for promoting paediatric LT, since paediatric recipients in the LE1 group achieve the largest health gains, approaching 30 LYG, approximately 40–45% higher than the adult recipients in the LE1 group (≈20 LYG) and nearly double of the adult recipients in the LE2 group (≈15 LYG). Consequently, the economic expenditure per life-year gained (i.e., the cost-effectiveness ratio in euros/LYG) is lower for paediatric than for adult recipients, freeing time and resources for a more efficient social and economic integration. In contrast, the cost-effectiveness ratio for adult recipients exhibits a clear plateau, remaining largely unchanged over the past 20 years.

## Discussion

The annual number of LTs performed in Spain is comparable to that of France or Italy; however, these countries have 50% more population. Consequently, considering the rate of LTs in relation to their population, Spain ranks fourth worldwide (with 26.44 LTs per million population), behind South Korea (30.78), Croatia (30.0) and US (27.03) [18]. Another distinguishing feature of Spain’s LT activity is its low rate of living donors per million population at 0.15, compared to the United Kingdom (0.49), China (0.66), US (1.76), India (8.02) and South Korea (17.79) [16]. Spain’s leadership in organ donation contributes notably to this ranking, but the ONT and the local donor management institutions also play a key role, as evidenced by data from other European organizations. For example, Scandiatransplant (which includes Denmark, Estonia, Finland, Iceland, Sweden, and Norway) reported 371 LTs with a median waiting time of 57 days in 2024 [34], while Eurotransplant (which includes Austria, Belgium, Croatia, Germany, Hungary, Luxemburg, the Netherlands, and Slovenia) performed 1,740 LTs in 2024, with 60% of candidates waiting 180 days during 2017–2021 [35]. In contrast, Spain reported 1343 LTs [17] with a median waiting time of 44 days in 2024 (see Table A3), which highlights the efficiency of its system. By demonstrating that a publicly funded system can achieve both clinical excellence and economic sustainability, the Spanish framework serves as a socioeconomic benchmark for the long-term viability of organ transplantation programs worldwide.

Some detailed studies have addressed and modelled LT cost in countries both larger and smaller than Spain [36–38]. In general, the cost of LT for administrative decision-makers depends on the prevalence of severe cases [37], the heterogeneity of the involved centers [39], as well as the availability of donors and the management of waiting lists [21]. With an annual budget of about €7 million [40], the ONT’s mission is to promote organ donation among the Spanish population and provide economic support to recipients’ families, medical staff involved in the transplant, and non-governmental organizations (NGOs) aligned with its objectives. The annual ONT budget can be considered part of the administrative costs for overall transplant activity (including liver, kidney, heart, etc.), as it ensures the availability of organs. Of this budget, the proportion allocated to LT represents at most a few million euros per year. Nevertheless, thanks to its guidelines and the strong coordination of the Spanish health system, the LT waiting list manages approximately 1,500–2,000 patients annually, maintaining an average of around 300 active patients at any given time. This framework has resulted in a sustained reduction in waiting times, which in some cases have fallen to as little as 2–3 weeks (see Table A4). The number of retransplantations, which account for less than 9% of all LT procedures according to RETH records (see Figure A2), serves as an indicator of severe cases. Each Spanish region has a network of LT centers, some of which are specialised in paediatric procedures, complemented by a logistics system that allows donor sharing among centers making a significant difference compared to health systems with smaller LT volumes [36]. In consequence, the heterogeneity of LT centers, as well as the management of donors and waiting lists were excluded from LT model cost.

Our estimates of annual economic income show that, even accounting for its corresponding fraction of ONT cost, LT activity generates at least one hundred million euros annually in the last years through the productivity of surviving recipients. This finding is relevant not only because it suggests that the LT activity tends toward economic sustainability, but also because it provides evidence of improvements in both the efficiency and the effectiveness of the health care system over time (including the normative framework, surgery staff and techniques, nursing healthcare and hospital protocols). The largest economic impact is generated by adult recipients aged 40–59 years (Group 3), who yield the highest economic return. Paediatric recipients (< 18 years, Group 1) also contribute substantially over the life course, as they transition into adulthood (Groups 2 and 3) and may even undergo a retransplantation in adulthood and thereby continuing to generate economic returns (Groups 3 and 4). On the other hand, older recipients (Group 4) exhibit a more limited economic contribution due to a shorter remaining life expectancy; nevertheless, as overall life expectancy continues to increase, their economic balance could also improve in the future. With our proposed model, alternative scenarios can easily be explored. For example, a retrospective evaluation could examine variations in the RW factor between age groups, or a prospective evaluation could assess the impact of significant changes in salary. Such factors may influence the current balance; however, their effects would become relevant only over time, particularly when the gap between healthcare investment and the income generated by recipients narrows or disappears. This is primarily driven by the sustained increase in the number of LTs and the rising life expectancy of recipients. Thus, the future sustainability of the transplant system depends on socio-economic and political support for transplant activity. If donor rates or investments in medical training diminish, or an imbalance across recipient age groups emerges, the system may quickly lose its economic equilibrium. At present, however, the system appears to exhibit positive inertia, which supports its continued growth and stability.

Surviving recipients often regain functional independence, which provides stability and reassurance within their immediate social environment, and re-engage in professional and cultural activities. As a result, both individual quality of life and broader societal well-being improve. Such outcomes demonstrate the broader societal value of LT programs, which extends well beyond direct clinical or economic benefits. LT also strengthens social cohesion and public trust in organ donation systems, highlighting the profound non-economic impact of transplantation on society.

Assuming a minimum of 16 LYG for an adult recipient, a gross estimation of the cost-effectiveness ratio for LT is around €4.77 thousand per life-year gained, calculated by dividing the combined cost of surgery and 16 years of immunosuppressive therapy by 16. Comparable cost-effectiveness profiles have also driven investment in related liver-disease diagnostics. For example, recent studies suggest that LiverMultiScan^1^ a commercial MRI-based software used to non-invasively characterize metabolic liver disease, reduces healthcare costs by avoiding biopsy procedures, thereby saving thousands of euros per year. It has also demonstrated a favourable incremental cost-effectiveness ratio of €4.92 thousand [41, 42], enabling its developers to secure €55 million in funding, including 18% from UK venture capital firms [43, 44]. Although not directly designed for transplantation, LiverMultiScan illustrates how economically efficient liver-assessment technologies can attract substantial investment, an insight that becomes relevant as comparable innovations emerge within the transplantation workflow. One such innovation is LiverColor^2^ [45, 46], an AI-powered software tool for intraoperative assessment of donor liver steatosis. By providing rapid, objective, and standardized quantification of steatosis at the point of procurement, LiverColor reduces reliance on subjective visual inspection (one of the main sources of misclassification leading to the unnecessary discard of viable organs [47, 48]). More accurate real-time evaluation allows transplant teams to confidently utilize grafts that might otherwise be rejected due to overestimated steatosis, thereby expanding the usable donor pool and improving the overall economic efficiency of transplantation. This is particularly relevant since healthcare systems confront rising demand and persistent scarcity of suitable donor organs. Therefore, tools that standardize and support intraoperative assessment, especially those deployable via low-cost digital infrastructure, offer high-leverage opportunities to enhance both clinical outcomes and long-term system sustainability. Beyond improving liver assessment, another critical factor in organ availability is preservation. Reducing warm ischemia (the time between organ retrieval and transplantation) directly impacts graft viability, and investments in devices [49, 50] or techniques [51, 52] that mitigate ischemic injury are therefore crucial.

### Limitations of the study

This study has several limitations, including reliance on aggregated historical data, missing patient-level information, and economic modelling assumptions that may not fully capture patient heterogeneity or indirect societal costs, thereby providing only an approximation of the average situation. While long-term economic contributions are assessed, non-economic impacts such as changes in quality of life and caregiver burden are not quantified. Additionally, trends over four decades may be affected by changes in data collection and reporting protocols within the responsible institutions. Despite these limitations, the study provides a comprehensive assessment of the economic value of LT in Spain over the past 40 years. By integrating diverse data sources, this framework reveals systemic trends and societal impacts that are not visible when these variables are analysed in isolation. This highlights the need for sustained investment in innovative solutions to improve patient outcomes, maximize the economic return of transplantation programs, and strengthen overall system sustainability.

## Conclusions

Over the past forty years, LT in Spain has evolved into a consolidated therapeutic option with profound medical, social, and economic impacts. With 52.6 donors per million population in 2024, Spain continues to lead Europe and maintain its global leadership in organ donation, while the increasing number of LTs reflects both the strength of its organizational model and the solidarity of its citizens. During this period, the number of transplants has stabilised at roughly 1,200 per year. The recipient profile is gradually shifting toward older adults, but recipients between 40 and 59 years of age still account for approximately 50% of all procedures. Advances in donor and recipient selection, surgical techniques, and post-transplant care have consistently improved patient survival. Economically, LT has generated a positive net contribution to Spanish mean income since the early 2000 s, underscoring its long-term value to society. These findings suggest that the Spanish transplantation system may serve as a benchmark for assessing the sustainability and societal impact of transplant programs in other health systems. Looking forward, the continued success of this model depends on its ability to integrate technological advances. Future policies should prioritize targeted funding for graft preservation and evaluation technologies, which are essential for reducing marginal costs, maximizing societal benefits, and reinforcing the model’s viability and relevance for the global transplantation community.

### Agreements

We would like to thank each of the people responsible for the Spanish Liver Transplant Registry (RETH) in the Liver Transplant Units: Dra. Laura Lladó (Hospital de Bellvitge), Dr. Itxarone Bilbao (Hospital Vall d’Hebron), Dr. José Luis Calleja (Hospital Puerta de Hierro Majadahonda), Dra. Ana Arias (Hospital Puerta de Hierro Majadahonda), Dra. Yiliam Fundora (Hospital Clínic), Dr. Carmelo Loinaz (Hospital 12 de Octubre), Dr. Félix Cambra (Hospital 12 de Octubre), Dr. Francisco Sánchez Bueno (Hospital Virgen de la Arrixaca), Dr. Antonio Poyato (Hospital Reina Sofía), Dr. Miguel Ángel Gómez Bravo (Hospital Virgen del Rocío), Dra. Magdalena Salcedo (Hospital Gregorio Marañón), Dr. Fernando Rotellar Sastre (Clínica Universidad de Navarra), Dr. Loreto Hierro (Hospital Infantil La Paz), Dra. Mar Achalandabaso Boira (Hospital Marqués de Valdecilla), Dra. Andrea Boscá (Hospital La Fe), Dr. Vicente Ibáñez Pradas (Hospital La Fe Infantil), Dr. Javier Nuño (Hospital Ramón y Cajal), Dr. Adolfo López Buenadicha (Hospital Ramón y Cajal), Dra. Alejandra Otero (CHU de A Coruña), Dr. Santiago Tomé Martínez de Rituerto (Hospital Clínico Univ. de Santiago), Dr. Mikel Gastaca (Hospital de Cruces), Dr. Ayaya Alonso Alvarado (Hospital Ntra. Sra. de la Candelaria), Dr. Julio Santoyo (Hospital Regional de Málaga), Dra. Sara Lorente Pérez (Hospital Clínico Univ. de Zaragoza), Dra. Carolina Almohalla Álvarez (Hospital Río Hortega), Dra. María Trinidad Villegas Herrera (Hospital Virgen de las Nieves), Dra. Carmen Bernardo (Hospital Central de Asturias), Dr. Gerardo Blanco Fernández (Hospital Univ. de Badajoz), Dr. José Manuel Ramia Ángel (Hospital Univ. Dr. Balmis), Dra. Lidia Sastre (Hospital Son Espases).

## Supplementary Information

Supplementary Material 1.

## Data Availability

The data that has been used is publicly available.

## Abbreviations

AP: Active Population
EPA: Encuestas a la Población Activa
LE1: Life Expectancy 1
LE2: Life Expectancy 2
LT: Liver Transplant
LYG: Life-Years Gained
MELD: Model for End-Stage Liver Disease
ONT: Organización Nacional de Trasplantes
RETH: Registro Español de Trasplante Hepático
RW: Return-to-work ratio
SETH: Sociedad Española de Trasplante Hepático
SR: Surviving Recipients

## Footnotes

1 https://www.perspectum.com/our-products/livermultiscan

2 https://www.upf.edu/en/web/mdm-dtic/projects1/-/asset_publisher/8JPTwPyOXW4y/content/livercolor
