# Supplementary material for: The Spanish transplantation model as a benchmark: clinical, societal, and economic impact of liver transplants in Spain (1984–2024)
Source: Health Econ Rev. 2026 Jun 2;16:92. doi: 10.1186/s13561-026-00801-4 (PMC13465184; doi:10.1186/s13561-026-00801-4)
Supplement: Supplementary file 1 [file 13561_2026_801_MOESM1_ESM.docx]

## The Spanish Transplantation Model as a Benchmark: Clinical, Societal, and Economic Impact of Liver Transplants in Spain (1984–2024)

Edson Plasencia Sánchez^1^, Concepción Gómez Gavara^2, *^, Glòria Merino Pinto^3^, Roger Sabater Mezquita^3,4^, Itxarone Bilbao^2,5^, Gerardo Blanco-Fernández^6^, Gloria de la Rosa^7^, Gemma Piella^1, *^

^1^ SIMBIOsys Group, Department of Engineering, *Universitat Pompeu Fabra,* 08018 Barcelona, Spain.

^2^ Department of Hepatic and Biliary Surgery and Transplants, *Hospital Universitari Vall d’Hebron*, 08035 Barcelona, Spain.

^3^ Centre de Recerca en Economia i Salut, Departament d'Economia i Empresa, *Universitat Pompeu Fabra*, 08005 Barcelona, Spain.

^4^ Department of Surgery, School of Medicine, *Universitat Autònoma de Barcelona*, 08193 Bellaterra, Spain

^5^ Department of Econometrics, Statistics and Applied Economics, *Universitat de Barcelona*, 08007 Barcelona, Spain

^6^ Sociedad Española de Trasplante Hepático (SETH), Spain.

^7^ Organización Nacional de Trasplantes (ONT), Ministerio de Sanidad, Spain.

^*^ Co-corresponding authors:

Concepción Gómez Gavara (concepcion.gomez@vallhebron.cat), *Hospital Universitari Vall d’Hebron*, *Pg. Vall d’Hebron* 119, 08035 Barcelona, Spain.

Gemma Piella (gemma.piella@upf.edu), *Universitat Pompeu Fabra, Carrer de Tanger* 122, 08018 Barcelona, Spain.

I. Recipient Life expectancy 1984-2023

Table A1 - Ratio between surviving and operated paediatric recipients over time. Overall data (paediatric and adult) from RETH 2005 and 2008, Cholestasis and Cirrhosis from RETH 2005, 2015 and 2023. “N.A.” stands for data not available.

| Surviving | Overall |  | Cholestasis | |  | Cirrhosis |  |  |
| --- | --- | --- | --- | --- | --- | --- | --- | --- |
| years | 1984-1987 | 1988-1990 | 1991-2005 | 2006-2015 | 2016-2023 | 1991-2005 | 2006-2015 | 2016-2023 |
| 1 | 0.560 | 0.771 | 0.876 | 0.925 | 0.943 | 0.750 | 0.724 | 0.824 |
| 2 | 0.516 | 0.713 | 0.870 | 0.919 | 0.940 | 0.718 | 0.687 | 0.775 |
| 3 | 0.472 | 0.654 | 0.864 | 0.913 | 0.936 | 0.686 | 0.650 | 0.726 |
| 4 | 0.463 | 0.632 | 0.858 | 0.909 | 0.933 | 0.654 | 0.650 | 0.726 |
| 5 | 0.453 | 0.609 | 0.852 | 0.905 | 0.929 | 0.622 | 0.650 | 0.726 |
| 6 | 0.444 | 0.595 | 0.842 | 0.901 | 0.926 | 0.593 | 0.619 | 0.713 |
| 7 | 0.435 | 0.581 | 0.831 | 0.897 | 0.922 | 0.563 | 0.588 | 0.700 |
| 8 | 0.426 | 0.568 | 0.821 | 0.893 | 0.919 | 0.534 | 0.556 | 0.686 |
| 9 | 0.416 | 0.554 | 0.810 | 0.889 | 0.915 | 0.504 | 0.525 | 0.673 |
| 10 | 0.407 | 0.540 | 0.800 | 0.885 | 0.912 | 0.475 | 0.494 | 0.660 |
| 11 | 0.402 | 0.523 | 0.800 | 0.883 | 0.910 | 0.475 | 0.474 | 0.651 |
| 12 | 0.400 | 0.514 | 0.800 | 0.881 | 0.908 | 0.475 | 0.454 | 0.642 |
| 13 | 0.397 | 0.506 | 0.800 | 0.879 | 0.905 | 0.475 | 0.435 | 0.634 |
| 14 | 0.395 | 0.497 | 0.800 | 0.877 | 0.903 | 0.475 | 0.415 | 0.625 |
| 15 | 0.395 | 0.497 | 0.800 | 0.875 | 0.901 | 0.475 | 0.395 | 0.616 |
| 16 | 0.360 | 0.486 | N.A. | N.A. | 0.897 | N.A. | N.A. | 0.616 |
| 17 | 0.326 | 0.474 | N.A. | N.A. | 0.893 | N.A. | N.A. | 0.616 |
| 18 | 0.291 | 0.463 | N.A. | N.A. | 0.889 | N.A. | N.A. | 0.616 |
| 19 | 0.257 | 0.451 | N.A. | N.A. | 0.885 | N.A. | N.A. | 0.616 |
| 20 | 0.222 | 0.440 | N.A. | N.A. | 0.882 | N.A. | N.A. | 0.616 |
| 21 | N.A. | N.A. | N.A. | N.A. | 0.878 | N.A. | N.A. | 0.616 |
| 22 | N.A. | N.A. | N.A. | N.A. | 0.874 | N.A. | N.A. | 0.616 |
| 23 | N.A. | N.A. | N.A. | N.A. | 0.870 | N.A. | N.A. | 0.616 |
| 24 | N.A. | N.A. | N.A. | N.A. | 0.866 | N.A. | N.A. | 0.616 |

Table A2 - Ratio between surviving and operated adult recipients over time. Overall data (paediatric and adult) from RETH 2005 and 2008, Cholestasis and Cirrhosis from RETH 2005, 2015 and 2023. “N.A.” stands for data not available.

| Surviving | Overall |  | Cholestasis | |  | Cirrhosis |  |  |
| --- | --- | --- | --- | --- | --- | --- | --- | --- |
| years | 1984-1987 | 1988-1990 | 1991-2005 | 2006-2015 | 2016-2023 | 1991-2005 | 2006-2015 | 2016-2023 |
| 1 | 0.560 | 0.771 | 0.849 | 0.893 | 0.903 | 0.830 | 0.859 | 0.874 |
| 2 | 0.516 | 0.713 | 0.835 | 0.876 | 0.885 | 0.796 | 0.826 | 0.843 |
| 3 | 0.472 | 0.654 | 0.822 | 0.858 | 0.867 | 0.762 | 0.793 | 0.812 |
| 4 | 0.463 | 0.632 | 0.808 | 0.851 | 0.859 | 0.728 | 0.768 | 0.788 |
| 5 | 0.453 | 0.609 | 0.794 | 0.843 | 0.851 | 0.694 | 0.742 | 0.763 |
| 6 | 0.444 | 0.595 | 0.777 | 0.828 | 0.836 | 0.669 | 0.718 | 0.738 |
| 7 | 0.435 | 0.581 | 0.760 | 0.813 | 0.820 | 0.643 | 0.694 | 0.713 |
| 8 | 0.426 | 0.568 | 0.742 | 0.798 | 0.805 | 0.618 | 0.671 | 0.688 |
| 9 | 0.416 | 0.554 | 0.725 | 0.783 | 0.789 | 0.592 | 0.647 | 0.663 |
| 10 | 0.407 | 0.540 | 0.708 | 0.768 | 0.774 | 0.567 | 0.623 | 0.638 |
| 11 | 0.402 | 0.523 | 0.701 | 0.747 | 0.753 | 0.551 | 0.600 | 0.614 |
| 12 | 0.400 | 0.514 | 0.695 | 0.726 | 0.732 | 0.535 | 0.578 | 0.590 |
| 13 | 0.397 | 0.506 | 0.688 | 0.706 | 0.711 | 0.519 | 0.555 | 0.565 |
| 14 | 0.395 | 0.497 | 0.682 | 0.685 | 0.690 | 0.503 | 0.533 | 0.541 |
| 15 | 0.395 | 0.497 | 0.675 | 0.664 | 0.669 | 0.487 | 0.510 | 0.517 |
| 16 | 0.360 | 0.486 | N.A. | 0.648 | 0.647 | N.A. | 0.488 | 0.494 |
| 17 | 0.326 | 0.474 | N.A. | 0.632 | 0.625 | N.A. | 0.467 | 0.470 |
| 18 | 0.291 | 0.463 | N.A. | 0.615 | 0.604 | N.A. | 0.445 | 0.447 |
| 19 | 0.257 | 0.451 | N.A. | 0.599 | 0.582 | N.A. | 0.424 | 0.423 |
| 20 | 0.222 | 0.440 | N.A. | 0.583 | 0.560 | N.A. | 0.402 | 0.400 |
| 21 | N.A. | N.A. | N.A. | 0.567 | 0.545 | N.A. | 0.380 | 0.379 |
| 22 | N.A. | N.A. | N.A. | 0.551 | 0.530 | N.A. | 0.359 | 0.358 |
| 23 | N.A. | N.A. | N.A. | 0.534 | 0.515 | N.A. | 0.337 | 0.338 |
| 24 | N.A. | N.A. | N.A. | 0.518 | 0.500 | N.A. | 0.316 | 0.317 |
| 25 | N.A. | N.A. | N.A. | N.A. | 0.475 | N.A. | N.A. | 0.296 |
| 26 | N.A. | N.A. | N.A. | N.A. | 0.450 | N.A. | N.A. | 0.275 |
| 27 | N.A. | N.A. | N.A. | N.A. | 0.425 | N.A. | N.A. | 0.242 |
| 28 | N.A. | N.A. | N.A. | N.A. | 0.400 | N.A. | N.A. | 0.225 |
| 29 | N.A. | N.A. | N.A. | N.A. | 0.375 | N.A. | N.A. | 0.208 |
| 30 | N.A. | N.A. | N.A. | N.A. | 0.350 | N.A. | N.A. | 0.225 |
| 31 | N.A. | N.A. | N.A. | N.A. | 0.350 | N.A. | N.A. | 0.200 |
| 32 | N.A. | N.A. | N.A. | N.A. | 0.350 | N.A. | N.A. | 0.200 |

Figure A1 – Ratio between surviving and operated adult (A panel) and paediatric (B panel) recipients over time between 1991 and 2023. Kaplan-Meier charts and legends taken directly from RETH 2023.

II. Re-transplant evolution over time (1984-2023)

Figure A2 – Evolution of re-transplant procedures over time 1984-2023. Lines: 1R.Tx., 2R.Tx. and 3R.Tx. represent the cumulative number of first, second and third re-transplant procedures (left axis). Line %R.Tx. represents the percentage of the cumulative re-transplant with respect of the cumulative number of transplants (right axis). Values before 1998 were interpolated.

III. Waiting time evolution over 2016-2023

Table A3 – Time (days) in Waiting list evolution over time 2016-2023. “N” and “IQR” stand for Number of patients and Interquartile range, respectively. Data from: *Memoria actividad donación y trasplante hepático* (Ministerio de Sanidad and ONT, 2020 and 2024)

| Year | N | Median (IQR) |
| --- | --- | --- |
| 2016 | 2115 | 121 (37-258) |
| 2017 | 1985 | 86 (21-200) |
| 2018 | 1829 | 60 (17-150) |
| 2019 | 1798 | 53 (15-131) |
| 2020 | 1628 | 76 (25-173) |
| 2021 | 1593 | 71 (21-166) |
| 2022 | 1608 | 55 (15-144) |
| 2023 | 1728 | 43 (12-117) |
| 2024 | 1822 | 45 (14-122) |

Table A4 – Time (days) in Waiting list 2023 per age group (excluding urgent and multivisceral cases). “N” and “IQR” stand for Number of patients and Interquartile range, respectively. Data from: *Memoria actividad donación y trasplante hepático* (Ministerio de Sanidad and ONT, 2020 and 2024).

|  | Overall | | Transplanted | |
| --- | --- | --- | --- | --- |
| Age | N | Median (ICR) | N | Median (ICR) |
| < 18 | 83 | 52 (13-115) | 65 | 47 (13-95) |
| 18-29 | 36 | 61 (15-121) | 24 | 68 (15-121) |
| 30-44 | 80 | 47 (22-120) | 51 | 44 (21-93) |
| 45-59 | 563 | 52 (18-127) | 410 | 41 (15-104) |
| 60-69 | 831 | 50 (21-119) | 529 | 45 (18-110) |
| ≥70 | 111 | 48 (16-75) | 85 | 35 (15-73) |
| Total | 1704 | 50 (19-119) | 1227 | 44 (16-105) |
